# Delayed reception of live blowfly (*Calliphora vicina* and *Chrysomya rufifacies*) larval samples: implications for minimum postmortem interval estimates

**DOI:** 10.1080/20961790.2017.1408550

**Published:** 2017-12-26

**Authors:** Melanie S. Archer, Stephanie D. Jones, James F. Wallman

**Affiliations:** a Department of Forensic Medicine, Monash University/Victorian Institute of Forensic Medicine, Southbank, Australia; b Centre for Sustainable Ecosystem Solutions, School of Biological Sciences, University of Wollongong, Wollongong, Australia

**Keywords:** Forensic science, forensic entomology, forensic evidence, larval development, best practice, sampling

## Abstract

Forensic entomology evidence collected by police and mortuary staff may be delayed in getting to the entomologist. Live samples may continue developing and alter minimum postmortem interval (PMI_min_) estimates. This study investigated development of simulated evidential samples of *Calliphora vicina* Robineau-Desvoidy and *Chrysomya rufifacies* (Macquart) (Diptera: Calliphoridae) maggots. Maggots of each species were studied in three developmental classes: “small” (late second/early third instar), “mid” (mid third instar) and “large” (late third instar). Seven replicates of 11 maggots in each class were assigned without food to four treatments: (1) 24 h at 20 °C; (2) 24 h at 4 °C; (3) 48 h at 20 °C and (4) 48 h at 4 °C. There was a significant difference in absolute length change across treatments, reflecting size for *C. vicina*, and interaction between size, time and temperature for *Ch. rufifacies*. *Calliphora vicina* maggots showed minimal mortality, and most second instar larvae moulted by the experiment's end. *Chrysomya rufifacies* showed heavy mortality and minimal moulting from the second to third instar. Only “large” *Ch. rufifacies* maggots kept at room temperature for 48 h pupariated. Since these results confirm that development can continue in live unfed maggot samples after their collection, it is not advisable to delay their preservation.

## Introduction

Impeccable evidence collection is at the heart of a solid forensic opinion, and failure to follow protocol can result in serious consequences in court [1,2]. There are various types of evidence collection problems, which include contamination, incomplete collection, incorrect sample handling (e.g. lack of secure and environmentally controlled storage conditions, inadequate chain of custody), deviation from accepted protocol (including use of non-validated or incorrect methods) and delayed delivery of perishable or live evidence to analysts. These problems have beset a variety of forensic science disciplines, prompting numerous papers that provide instructions for optimal evidence collection and training standards, thereby outlining “best practice” [3–6].

Situations occasionally arise where forensic entomologists are faced with a sub-optimal evidence collection. They are often not in a position to collect their own evidence, or may be consulted about a case after the time for optimal evidence collection has passed. Entomology evidence, however, needs to be collected early, and requires specialist knowledge for its collection because the techniques used must be applied precisely. Problems with collection techniques arise because crime scene or mortuary personnel may be inadequately trained, too busy with other investigative tasks, or do not realize until too late that they need an entomology opinion. Additionally, proxy collectors are only infrequently asked to collect entomology evidence, and may therefore be prone to mistakes caused by inexperience.

Sub-optimal evidence collection can be combatted with frequent collector education and a close working relationship with proxy collectors and death investigators. However, despite best efforts, problems can occur in any jurisdiction. Research that describes the consequences of sub-optimal evidence collection is therefore critical because it allows the entomologist to quantify and predict its effects. In the worst case scenario, they may have to abandon estimating minimum postmortem interval (PMI_min_), or at best, determine that the deficiency will have minimal effect. Some direct research has been performed about the consequences of poor evidence collection, and the results of some general forensic entomology research can also be used to infer the effects of poor evidence collection on case results. The effects of larval length reduction and discolouration in various non-standard preservative solutions have been measured [7–9]. Maggot post-feeding dispersal has been measured to show the importance of an adequate death scene soil search [10–12]. Case studies and research data have also been presented to elucidate the risks of poor evidence collection producing contamination of death scene [13,14] and mortuary [15] samples.

Timely entomological evidence collection is particularly important because most evidence is alive and may escape even before it is collected (especially following body disturbance). Evidence is also often in the form of growing juveniles that will continue to develop if not preserved. In particular, a preserved sample of maggots is ideally collected early in the death scene investigation to “stop the clock” on their development. This is best done early to minimize the complications of reconstructing the temperatures at which the maggots have grown.

The necessity of early evidence collection has been reinforced by the finding that maggot masses of various blowfly species can produce significant heat in mortuary fridges [16], which may allow growth and development above the refrigerator temperature [17]. While such continued maggot feeding can potentially affect PMI_min_, it also has scope to complicate matters for the pathologist by causing tissue destruction [18]. The immatures of some cold-adapted species have also been shown to develop at temperatures of ≤5 °C [19–22]. Much work in this area has focussed on feeding maggots that remained on their food source throughout experiments, and also on the effect of chilling on subsequent development, rather than on delineating what occurs during the period of refrigeration itself [19,23].While these results have alerted workers to be cautious with refrigerated and fed evidential samples, there has been no work to date describing whether feeding maggots (i.e. non pre-pupae) that have been removed from their food source and collected as a live sample grow or develop in the absence of food.

“Best practice” collection protocol stipulates that a properly preserved sample is collected as soon as possible, ideally at the site of body discovery [5], and the entomologist preferentially uses this sample to calculate the PMI_min_. However, there are sub-optimal situations where the entomologist has only a live maggot sample and has no choice but to preserve it for calculating the PMI_min_. These situations occur when the preserved sample has been incorrectly prepared, when collectors are unsure of how to prepare the preserved sample or unaware of the need to do so, when the preserved sample has been lost, or when there has been collection of an inadequately small preserved sample that needs additional maggots to give statistical rigour in deriving an age estimate.

It may be considered reasonable to assume that a live sample kept with no food will suspend growth and development, especially if refrigerated at low temperatures, and would therefore be interchangeable with a sample preserved early in the investigation. There is limited literature to describe whether growth and development (including instar change) can occur in unfed live evidential samples, either under refrigeration or at room temperature. Ždárek and Sláma [24] found that development of *C. vomitoria* larvae was suspended when they were starved from the start of the third instar, even for several days, but resumed when they were fed for as little as a few hours. There is also very little in the literature to suggest that a live sample cannot be substituted if necessary by preserving it upon receipt of the evidence. Smith [25] mentions that a live larval sample may have “aged” since collection, and should be regarded as separate to the preserved sample. However, it is not apparent whether this is only when food is provided, or whether aging constitutes size increase and/or instar change.

By convention, evidential samples of live maggots are placed into ventilated jars, with or without food depending on the jurisdiction, and/or with moistened paper to keep them hydrated [5,25,26]. They are then usually refrigerated at <6 °C to slow their metabolic processes [5], although some workers have recently cautioned against chilling specimens at temperatures near their development threshold [27]. There may be a delay between the collection of samples by scenes of crime or mortuary technicians, and their receipt by the entomologist, although it is recommended that this be kept below 24 h [5]. But if a live unfed sample does continue to develop, either under refrigeration or at room temperature, the entomologist risks overestimating the PMI_min_. Conversely, the live sample may lose mass and shrink if kept without food, leading to a risk of underestimating the PMI_min_ by preserving live samples too long after collection.

This study measured whether size increase and physiological development (crop emptying and instar change) occur when unfed live maggot samples of different ages are kept at room temperature or refrigerated. Mortality was also measured. Two forensically important cosmopolitan species were used to assess differing responses to storage conditions and provide the first data on the consequences of delays in reception of live unfed samples. We selected the cold-adapted *C. vicina* and the heat-adapted *Ch. rufifacies*. Both species are common in forensic casework in south-eastern Australia.

## Materials and methods

### Fly cultures

Experiments involving *C. vicina* were carried out in the forensic entomology laboratory of the Victorian Institute of Forensic Medicine (VIFM), and trials using *Ch. rufifacies* were conducted in the Forensic Entomology Research and Analysis Laboratory at the University of Wollongong (UOW). Standard parameters between both laboratories were temperature (20 °C) and light regimes (12:12 light/dark). Both cultures originated from the stocks of UOW. *Calliphora vicina* were an F13 generation from Silverdale, New South Wales (33°56’32” S, 150°34’48” E), and *Ch. rufifacies* were an F15 generation from Wollongong, New South Wales (34°24’18” S, 150°52’19” E).

Fly colonies were kept in plastic boxes (VIFM dimensions: 15 cm × 25 cm × 30 cm; UOW dimensions: 25 cm × 28 cm × 41 cm) with mesh sides and curtain material sleeves for access. Colonies were provided with minced kangaroo meat (VIP Pet Mince) to facilitate ovarian maturation, and sugar cubes and water *ad libitum*. Maggots for the experiments were obtained by first allowing colonies to oviposit onto a small portion of kangaroo mince. Approximately 300 eggs were then placed on 350 g kangaroo mince (excess to the feeding requirements of the larvae) in 850 mL round plastic containers and incubated at 20 °C.

### Baseline development and experimental treatments

There were three treatment age groups, and 20 maggots per age group were hot water killed and fixed and placed in 80% ethanol at the beginning of the replicate set-up in order to establish their baseline size and instar status. The three age groups constituted: “small” (peri-transitional between second and third instars), “mid” (mid third instar) and “large” (late third instar; Tables 1 and 2). All maggot age groups were feeding, except for “large” *Ch. rufifacies*, of which some were probably pre-pupae at the beginning of the experiment, although they were still located on their food source and had full crops. Baseline maggots were dissected under a stereo microscope to confirm that their crops were full.

**Table 1.** Baseline mean length ± SD (mm) and age (h) of *Calliphora vicina* larvae used for four treatments (*n* = 20).

Size class	Instar (proportion in instar)	Mean length ± SD (mm)	Age (h)
Small	Second (0.5), Third (0.1), Second/Third (0.4)	8.1 ± 1.0	72
Mid	Third (1.0)	14.1 ± 0.9	96
Large	Third (1.0)	19.2 ± 0.8	120

Four treatments were used: 24 h at 20 °C; 24 h at 4 °C; 48 h at 20 °C; and 48 h at 4 °C.

**Table 2.** Baseline mean length ± SD (mm) and age (h) of *Chrysomya rufifacies* larvae used for each treatment (*n* = 20).

Size class	Treatments	Instar (proportion in instar)	Mean length ± SD (mm)	Age (h)
Small	24 h 20 °C, 48 h 20 °C 24 h 4 °C	Second (1.0) Second (1.0)	5.7 ± 0.7 7.6 ± 0.7	72 72
	48 h 4 °C	Second (0.65), Third (0.35)	8.2 ± 0.5	72
Mid	24 h 20 °C, 48 h 20 °C 24 h 4 °C	Third (1.0) Third (1.0)	12.5 ± 2.3 13.4 ± 1.1	120 120
	48 h 4 °C	Third (1.0)	14.9 ± 1.0	120
Large	24 h 20 °C, 48 h 20 °C 24 h 4 °C	Third (1.0) Third (1.0)	13.9 ± 3.0 14.8 ± 0.9	168 168
	48 h 4 °C	Third (1.0)	16.3 ± 0.9	168

Preserved maggots were measured using a steel ruler to the nearest 0.5 mm within 12 h, both for the age group baseline calculation and for the experimental replicates. A separate experiment was done to ensure that no shrinkage occurred in the 80% ethanol over 12 h. Twenty larvae for each class size of *C. vicina* and *Ch. rufifacies* were obtained by growing them at the same times, temperatures and densities as for the main experiment (described below). They were killed by fixation in boiling water and then measured at times 0, 6 and 12 h using a steel ruler (measured to the nearest 0.5 mm) after preservation. Maggots of both species fixed at 0, 6 and 12 h for all sizes (“small”, “mid” and “large”) showed no significant length differences (Table 3).

**Table 3.** Demonstration that there is no maggot shrinkage in preservative over 12 h for *Calliphora vicina* or *Chrysomya rufifacies* (*n* = 20).

		6 h		12 h	
Species	Size class	*t*	*P*	*t*	*P*
*C. vicina*	Small	2.08	0.051	1.46	0.16
	Mid	0.31	0.76	0.46	0.65
	Large	−0.60	0.55	0.27	0.79
*Ch. rufifacies*	Small	0.71	0.49	0.59	0.56
	Mid	−1.68	0.11	−1.24	0.23
	Large	1.01	0.33	1.00	0.28

*t* provides the t-statistic comparing change in length of 20 maggots from 0 h.

### Maggot treatments

Each replicate simulated a live evidential sample, and consisted of 11 maggots each (either *C. vicina* or *Ch. rufifacies*). There were seven replicates per treatment per species for each of the three age groups. Eleven maggots and 7 replicates were used to enhance statistical rigour beyond that provided by multiples of 10 and 5, respectively. Each replicate was randomly assigned to one of four treatments, accomplished by coding the jars and mixing them up, then assigning maggots to jars with the labels obscured. The four treatments were: (1) 24 h at 20 °C; (2) 24 h at 4 °C; (3) 48 h at 20 °C; and (4) 48 h at 4 °C.

Replicates were prepared by removing maggots from their culture meat with forceps and placing them into 70 mL pathology specimen jars. No food was provided in order to simulate an unfed live evidential sample. Each replicate was kept hydrated throughout the experiment with a 6 cm × 6 cm piece of moistened tissue paper (these were all checked at the end of the experiment to ensure that they were still wet). A piece of curtain material affixed with an elastic band was placed over the mouth of each specimen jar to prevent the escape of maggots. All replicates were kept in the dark throughout the experiment to ensure that maggots were not excessively active due to attempts to avoid the light.

### Sample processing

At the end of each treatment (either 24 h or 48 h after set-up), mortality was recorded. Living maggots were killed and fixed in hot water and preserved in 80% ethanol, and were measured within 12 h of preservation with a steel ruler. The measurements for *C. vicina* were done blind to temperature (i.e. 20 °C or 4 °C) for replicates of the same age group and time by obscuring the replicate code until after all measurements had been finished. Blind measurement was not possible logistically for different times due to the 12 h limitation on measurement. It was also not possible for *Ch. rufifacies* due to a paucity of maggots limiting the simultaneous running of replicates. The instar of all maggots was recorded at the end of the experiment, and they were dissected under a stereo microscope in order to classify the crop as full or empty.

### Temperature control and measurement

ICod (Thermodata, Australia) temperature loggers were placed with each treatment throughout their duration to ensure that lab and fridge temperatures remained within ±1 °C. Temperatures of 20 °C were achieved in the open laboratory of VIFM and in the fly growth room at UOW. Temperatures of 4 °C were achieved in the mortuary refrigerator at VIFM and a Thermoline (Thermoline Scientific, Australia) growth chamber at UOW. The VIFM mortuary fridge was in operation at the time of the experiment, and although this meant that it was opened multiple times per day, the temperature remained stable due to the conduction of the experiment well away from the door. The opening of the fridge also simulated reality.

### Statistics

Species data were analysed separately, and univariate general linear models were constructed in SPSS Statistics Version 20 (IBM Corp.). The level of significance (α) was set at 0.05 for all tests. A one-way ANOVA was initially performed on data for the mean baseline length of *Ch. rufifacies* larvae to ensure a significant difference between the designated “small”, “mid” and “large” size classes. The results were further delineated with *posthoc* (Sheffé tests) to confirm that the difference was between each size class. These tests were performed due to variation within each size class that was caused by the use of different baseline stocks. Three-way ANOVA with size class (“small”, “mid” and “large”), time (24 h *vs*. 48 h) and temperature (fridge 4 °C *vs*. room 20 °C) as factors were used to determine absolute length changes between baseline maggots and maggots collected at the end of the experiment. The same model was used to investigate mortality for each species. Three-way ANCOVA (with mean final maggot length per replicate as the covariate) was used to analyse differences in proportions of maggots (transformed by arcsin of the square root) with empty crops between treatments and size classes.

## Results

### Development of live evidential maggots

**Figure 1.**
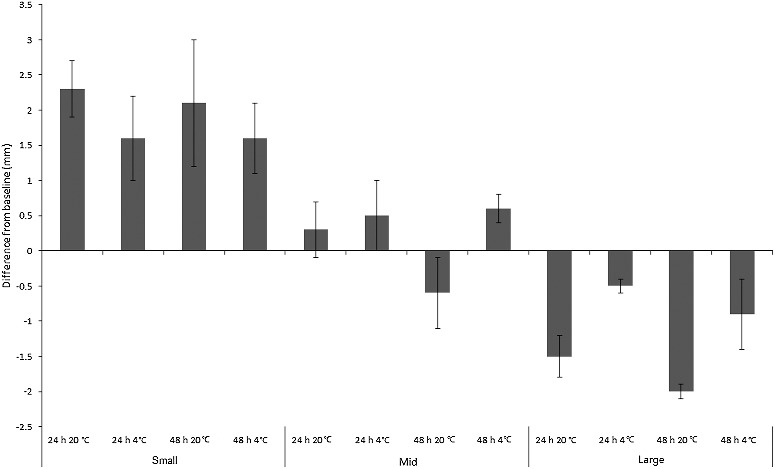
Mean change from baseline size (mm) of “small”, “mid” and “large”-sized *Calliphora vicina* maggots (*n* = 11 per replicate) in simulated evidence collection jars. Groups of *n* = 7 jars per treatment.

Development was manifested as length and/or instar change, which both occurred widely across treatments for both species. ANOVA revealed a significant difference in absolute length change across treatments for *C. vicina* (*F*_2,11_ = 17.8, *P* < 0.001), reflecting maggot age class (*F*_2,11_ = 67.9, *P* < 0.001). Length interacted with temperature, generally increasing more at 20 °C (*F*_2,11_ = 27.9, *P* < 0.001) but not over 48 h *vs*. 24 h (*F*_2,11_ = 2.7, *P* = 0.107), indicating that the most rapid length change occurred early. All *C. vicina* treatments showed a small degree of mean absolute length change from baseline during the experiment. However, the pattern depended on size class. Mean length change for “small” maggots was +1.9 mm and “large” maggots shrank by mean −1.2 mm. “Mid”-sized maggots grew overall by mean +0.2 mm, but actually reduced their length when kept at room temperature for 48 h (Figure 1).

The mean absolute length change results were not so clear cut for *Ch. rufifacies*. Most of the individual treatments were derived from multiple baseline stocks due to the logistics of breeding large numbers of larvae from the UOW colony simultaneously (Tables 1 and 2). This meant that even within the same size class, maggots of different treatments were derived from different baseline stocks, and had slight differences in their sizes despite being the same age (Tables 1 and 2). Also, there was some overlap in the mean sizes between the “mid” and “large” size classes. However, a one-way ANOVA between “small”, “mid” and “large” baseline maggot mean length confirmed a significant difference between the baseline lengths of the size classes (*F*_2,179_ = 334.9, *P* < 0.001). *Posthoc* (Sheffé tests) further confirmed that the significant differences existed between every size class (all with *P* < 0.001), thus correctly delineating them as “small”, “mid” and “large”.

**Figure 2.**
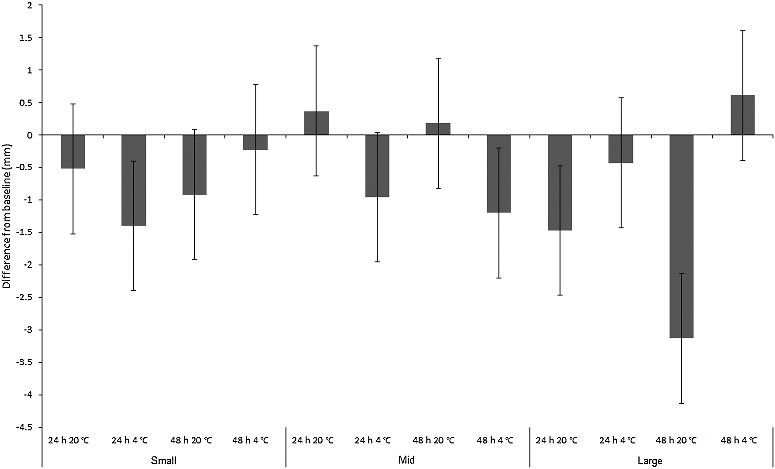
Mean change from baseline size (mm) of “small”, “mid” and “large”-sized *Chrysomya rufifacies* maggots (*n* = 11 per replicate) in simulated evidence collection jars. Groups of *n* = 7 jars per treatment.

The results for *Ch. rufifacies* were also affected by the heavy mortality within refrigerated “mid” and “small” size treatments, which meant that much of the measured length change was from dead larvae that did not survive the duration of the experiment. Nevertheless, if the practitioner has no alternative samples to choose from, they may be forced to consider preserving dead or moribund larvae for a PMI_min_ estimate, so the experiment should be considered representative of real-life conditions. ANOVA revealed a significant difference in absolute length change across treatments (*F*_2,11_ = 17.1, *P* < 0.001), reflecting maggot size class interacting significantly with time and temperature (*F*_2,11_ = 6.3, *P* < 0.001; Figure 2). Overall, mean length change for “small” *Ch. rufifacies* maggots was −0.8 mm, and for “mid”-sized maggots was −0.4 mm, and for “large” maggots was −1.1 mm. There was, however, a length increase in several treatments: “mid”-sized maggots at room temperature, and “large” maggots in the fridge for 48 h (Figure 2).

**Figure 3.**
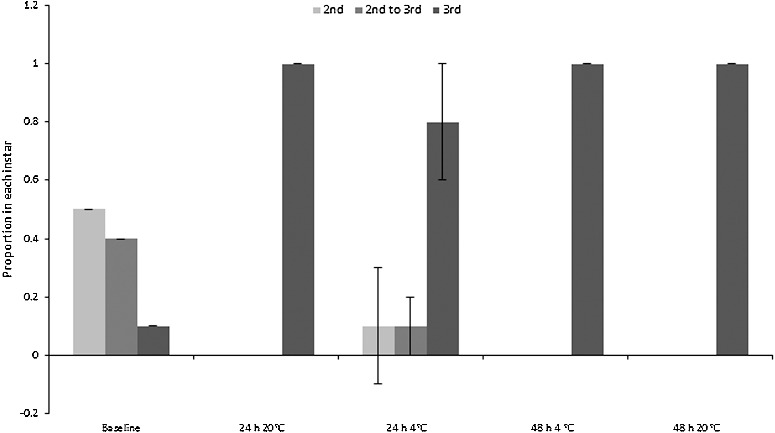
Proportions (± SD) of “small” *Calliphora vicina* maggots in the second and third instars, and transitional between second and third instars. Baseline was a pooled sample, so has no SD. The “mid” and “large” maggot size classes were all in the third instar at the start of the experiment, so are not depicted.

**Figure 4.**
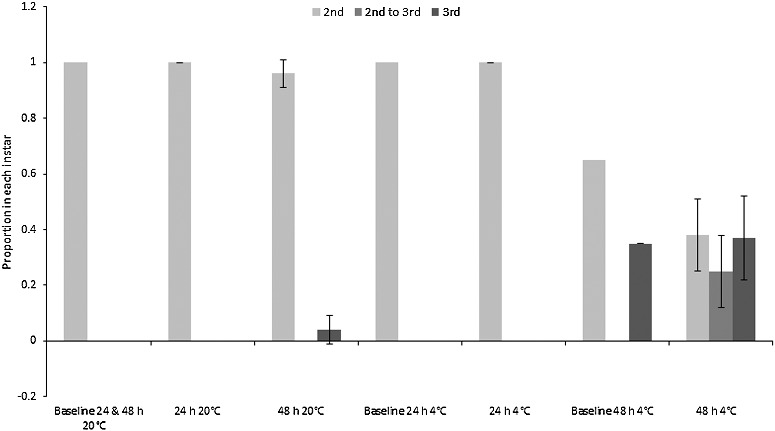
Proportions (± SD) of “small” *Chrysomya rufifacies* maggots in the second and third instars, and transitional between second and third instars. Baselines were pooled samples, so have no SD. The “mid” and “large” maggot size classes were all in the third instar at the start of the experiment, so are not depicted.

The majority of “small” *C. vicina* maggots moulted into their third instar during the experiment, therefore continuing their physiological development. Moulting could only be assessed for the “small” maggots because the “mid” and “large” size classes were already in their third instar at the beginning of the experiment, although no third instar *C. vicina* maggots pupariated. All “small” maggots eclosed to third instar in both of the 48 h treatments, and in the 24 h room temperature treatment. The proportion of third instar larvae also increased from mean 0.1 at baseline to mean (0.8 ± 0.2) in the 24 h fridge treatment (Figure 3). By contrast, the “small” *Ch. rufifacies* larvae showed minimal change from the second to third instar, although some moulting occurred within the 48 h fridge and room treatments (Figure 4). Pupariation of “large” *Ch. rufifacies* larvae also occurred in the 48 h room temperature treatment for a mean proportion of (0.5 ± 0.2) of larvae; no pupariation occurred in any other treatment.

### Crop status

**Figure 5.**
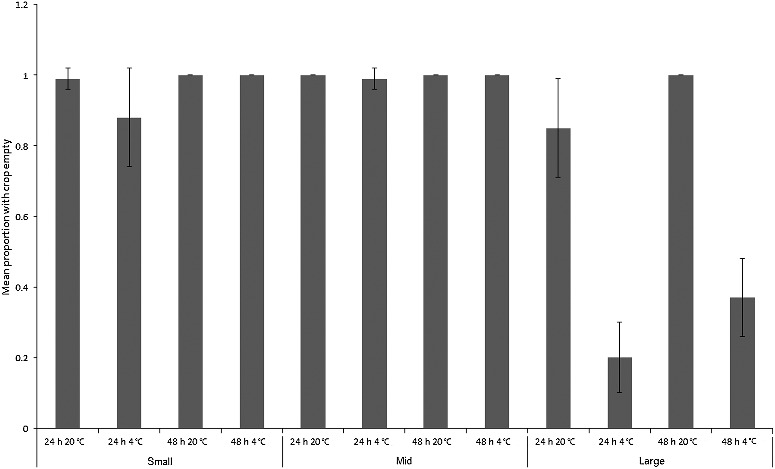
Mean proportion (± SD) of “small”, “mid” and “large” *Calliphora vicina* maggots with crops empty after each treatment (*n* = 7). All maggots in the baseline samples had full crops (still feeding).

**Figure 6.**
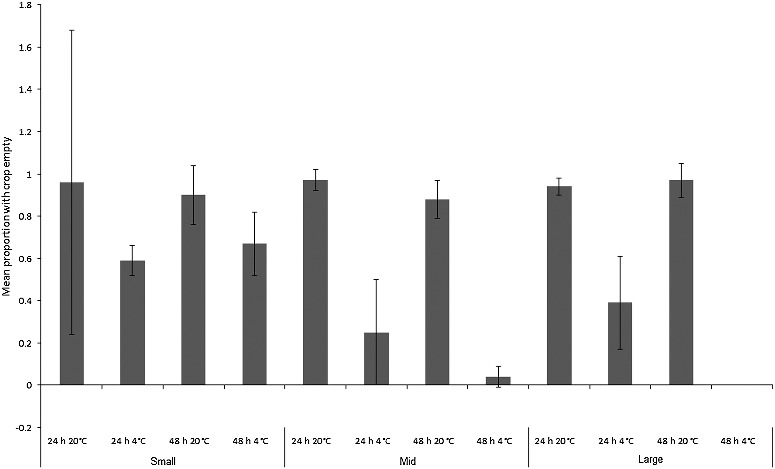
Mean proportion (± SD) of “small”, “mid” and “large” *Chrysomya rufifacies* maggots with crops empty after each treatment (*n* = 7). All maggots in the baseline samples had full crops (still feeding).

Degree of crop emptying at the end of the experiment was used as an indication of gut activity during the sample storage period. All baseline maggots were on their food source at the time they were sampled, and had full crops. However, the degree to which crops were emptied varied between treatments and species (Figures 5 and 6). A three-way ANCOVA was constructed for each species to examine differences between treatments in proportions of maggots with their crops empty (proportions transformed by arcsin of the square root), with mean final length (mm) of maggots per replicate as the covariate.

For *C. vicina*, the covariate was not significant (*F*_1,71_ = 0.6, *P* = 0.4). The overall model was highly significant (*F*_1,12_ = 59.3, *P* < 0.001), with size class (*F*_2,71_ = 83.0, *P* < 0.001), time (*F*_1,71_ = 24.9, *P* < 0.001) and temperature (*F*_1,71_ = 133.0, *P <* 0.001) being individually significant factors. There were significant interactions between size and time (*F*_2,71_ = 6.0, *P* < 0.001), and between size and temperature (*F*_2,71_ = 76.3, *P <* 0.001), but not between time and temperature (*F*_1,71_ = 0.9, *P* = 0.356), or between size and time and temperature (*F*_2,71_ = 3.0, *P* = 0.054; Figure 5). Digestive contents moved out of the crops of most maggots during the experiment, regardless of their size (Figure 5). The exception was “large” maggots kept in the fridge, which showed low rates of crop emptying. These two treatments showed correspondingly low rates of absolute length change (Figure 1), but, by contrast, the “mid”-sized maggots, which also showed low rates of absolute length change, emptied their crops.

A separate three-way ANCOVA for *Ch. rufifacies* revealed that the overall model was highly significant (*F*_1,12_ = 53.8, *P* < 0.001). The covariate of mean final maggot length per replicate was not significant (*F*_1,71_ = 0.8, *P* = 0.1), but there was a significant interaction between size class, time and temperature (*F*_2,71_ = 10.6, *P* < 0.001; Figure 6). Maggots at room temperature for both time intervals and of all size classes usually emptied their crops. Refrigerated maggots had much lesser degrees of crop emptying, especially for “mid” and “large” maggots, and especially for the 48 h treatments (Figure 6). It is counterintuitive that the 48 h fridge treatments for “mid” and “large” maggots should be more likely than 24 h maggots to have full crops, but these maggots all came from different baseline stocks, which may account for the difference.

### Mortality

**Figure 7.**
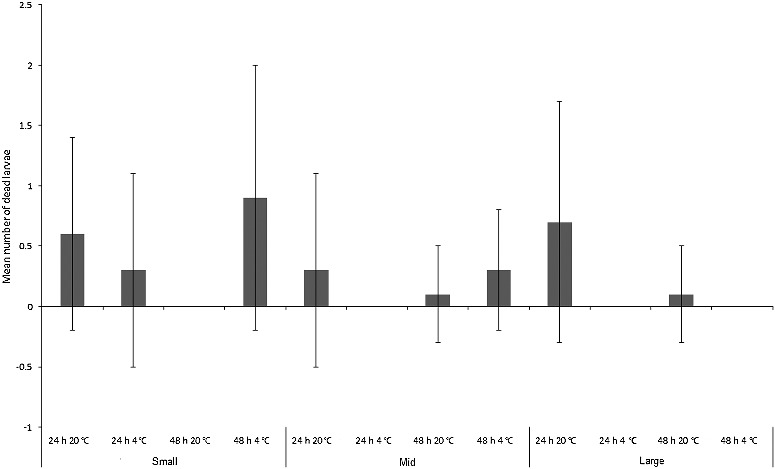
Mean number (± SD) of dead *Calliphora vicina* larvae in each size class (“small”, “mid” and “large”) for each treatment (*n* = 7).

The number of *C. vicina* larvae that died during the experiment was less than a mean of one larva per replicate across the experiment (Figure 7), and were thus not in sufficient numbers to provide larvae with a food source that could influence growth. All dead larvae were cannibalized by the remaining living maggots, but it is unknown whether this was the actual cause of death. The three-way ANOVA model with time, size and temperature incorporated was not significant overall (*F*_1,11_ = 1.7, *P* = 0.09), and there was no significant difference between mean number of larvae dying per treatment between size classes (*F*_2,71_ = 1.4, *P* = 0.2), between 24 h *vs*. 48 h (*F*_1,71_ = 0.3, *P* = 0.6) or between room *vs*. fridge temperature treatments (*F*_1,71_ = 0.3, *P* = 0.6; Figure 7).

**Figure 8.**
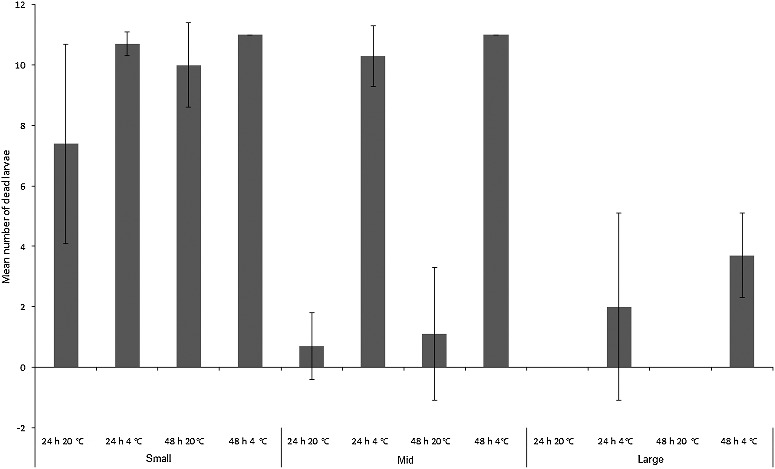
Mean number (± SD) of dead *Chrysomya rufifacies* larvae in each size class (“small”, “mid” and “large”) for each treatment (*n* = 7).

*Chrysoma rufifacies* larvae suffered far greater mortality than *C. vicina*. The three-way ANOVA model incorporating size class, time and temperature was significant overall (*F*_1,11_ = 60.7, *P* < 0.001) and there were significant differences between mean number of larvae dying per treatment between size classes (*F*_2,71_ = 185.1, *P* < 0.001), between 24 h *vs*. 48 h (*F*_1,71_ = 7.2, *P* < 0.05) and between room *vs*. fridge temperature treatments (*F*_1,71_ = 191.2, *P* < 0.001; Figure 8). There was also a significant interaction between size and temperature (*F*_2,71_ = 122.3, *P* < 0.001), mainly reflecting the fact that “large” larvae were less vulnerable to refrigeration than the “mid” and “small” size classes. Virtually all larvae from the “small” size class died regardless of treatment. “Mid”-sized larvae from the refrigerator treatments mostly died, whereas survival at room temperature was greatly improved. The only larvae to die in the “large” size class were from refrigerated treatments, although at a greatly reduced rate to those from the “small”- and “mid”-sized classes (Figure 8).

**Figure 9.**
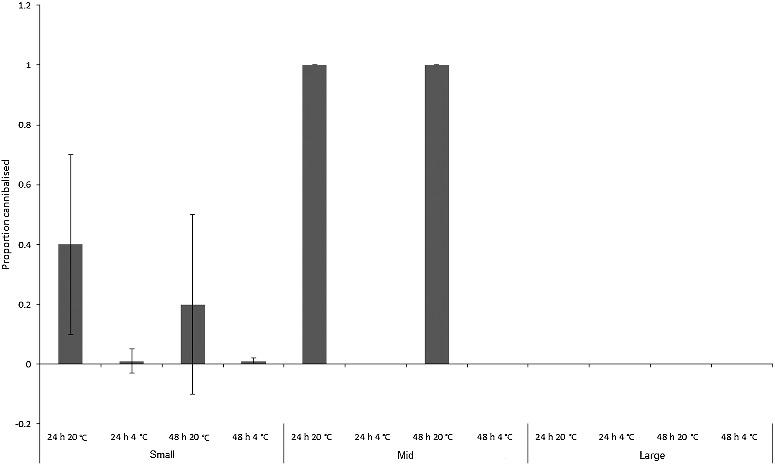
Mean proportion (± SD) of cannibalized *Chrysomya rufifacies* larvae in each size class (“small”, “mid” and large’) for each treatment (*n* = 7).

Many of the dead *Ch. rufifacies* larvae were cannibalized (Figure 9), although no statistics could be performed comparing cannibalism between treatments. This was due to the gaps in the mortality data-set produced by the two room temperature “large” larva treatments where all larvae survived, and the fact that few larvae in several of the other treatments died. Inspection of Figure 9 shows that rates of cannibalism increased greatly at room temperature, and peaked for the “mid”-sized treatments. There was no cannibalism seen in the “large” treatments. Cannibalism obviously provides a larval food source, and the two room temperature “mid”-sized treatments in which growth occurred (Figure 2) had high rates of cannibalism (Figure 9). However, the actual number of larvae that died was low in these treatments (Figure 8).

## Discussion

Simulated evidential maggots of both species and all size classes underwent some form of development in most treatments, and not even refrigeration, removal from food and short storage durations (24 h) prevented this. The ability of “small” *C. vicina* to gain length, and for “small” maggots of both species to moult, even after prolonged removal from their food source, is particularly surprising.

“Small” *C. vicina* maggots grew from baseline length, whereas “large” maggots decreased in length, especially at room temperature. “Mid”-sized *C. vicina* maggots changed minimally from baseline. But despite the development measured here, storage without food as live evidential samples for “small”- and “mid”-sized *C. vicina* larvae still greatly slows development, even without refrigeration. For example, if small larvae were maintained with food for 48 h at 20 °C, they would have roughly doubled their size [28], as opposed to the mean gain of only approximately 2 mm seen here. In real terms, a 2 mm length increase translates to less than a 12 h error, and in an appropriately conservative casework estimate, may even be encompassed by the length variation seen in natural maggot cohorts.

Size increase or decrease for this species could be explainable in terms of the expected “developmental programme” of each size class. “Small”- and “mid”-sized maggots were still growing, and can be expected to do so even if removed from their food source. It is possible that they were enabled to continue developing by the food still contained within their guts and fat reserves; crop emptying was widespread, and especially prevalent for the “small”- and “mid”-sized treatments. “Large” maggots, by contrast, were on the cusp of becoming pre-pupae, and shrinkage is expected once they are removed from their food source. It is in keeping with the results of Davies and Ratcliffe [21] for *C. vicina* that pre-pupae continue development under refrigeration. The shrinkage seen for “mid”-sized maggots after 48 h at room temperature could be an end result of initial growth (as seen for the other “mid”-sized treatments), followed by transformation to the pre-pupa when food continues to be withheld. This result is unlikely to be simple mass loss because comparison with “small” maggots, which should also have lost length if this were the case, shows that they did not reduce their length even after 48 h at room temperature.

The overall numbers of *C. vicina* third instar maggots in the “small” size class changed from very few at baseline to almost all maggots by the experiment's end. The entire size class transitioned to third instar for all but the 24 h refrigerated specimens, and it is likely that the cold temperature and short treatment duration are responsible for the lower moulting rate. The continuation of moulting demonstrates that the hormonal, behavioural and physiological processes that control moulting continued for the simulated evidential samples. It is also likely that many maggots were physiologically committed to moult due to being in the late second instar at the beginning of the experiment. Indeed, 40% were transitional between the second and third instars at baseline (apolysis had occurred and the pharate third instar maggot was visible). Moulting during evidential storage could be an important source of error when using data such as those of Anderson [29], which is aggregated by instar: change between instars will alter the upper and lower limits of developmental times within which a maggot's growth can be inferred to sit. In practical terms, “small” *C. vicina* larvae from this study were 72 h old at the beginning of the experiment, but if sampled at the end of the experiment with the assumption that no development had occurred during storage, then they would be aged according to Anderson's data [29] as being between at least (84 ± 10) h and (93.5 ± 0.5) h old.

All “small” *C. vicina* maggots were derived from the same baseline stock, so it is easy to compare the degree of development between treatments. By contrast, logistics dictated that the “small” *Ch. rufifacies* maggots were derived from three different baseline stocks, which were all at slightly different degrees of development, despite the same growth time and conditions. Therefore, the treatment results are not as directly comparable with each other as for *C. vicina*. Nevertheless, some moulting to third instar was recorded in both 48 h treatments (20 °C and 4 °C), so the important point is proven that *Ch. rufifacies* maggots of appropriate age can also transition between instars both in and out of the refrigerator. It is also pertinent to note that the moulting occurred despite the fact that there were no survivors from the refrigerated treatment, and virtually none from the room temperature treatment. This implies either that moulting occurred early on, or that it can occur even if larvae are moribund.

Crop emptying was also commonly recorded for both species, although all maggots at baseline had full crops. The degree of crop emptying seen upon dissection can be used to help distinguish third instar maggots from pre-pupae in preserved samples [20]. It has also been suggested that crop emptying for pre-pupal *C. vicina* occurs in a predictable manner that can allow aging of pre-pupae. Rapid discharge of contents occurs in the first day after feeding ceases, with crop length reducing to less than half. This is followed by gradual emptying over the days leading to pupariation [30]. *Chrysomya rufifacies*, by contrast, is said to empty its crop in a more gradual fashion [30].

The “large” *C. vicina* maggots in this experiment, as well as most “large” *Ch. rufifacies*, were definitely not quite pre-pupae at the time of separation from their food, which may be why their crop emptying patterns did not follow the patterns described above. Instead, “large” larvae of both species underwent rapid and usually total discharge of food from their crops within 24 h, unless refrigerated. There has been some research also with *C. vicina* on crop emptying pre-pupae chilled at 3 °C for 24 h [20], which were found not to empty their crops during the period of refrigeration, unlike those in the current experiment kept at 4 °C.

The results of this experiment show that while the crop dissection technique may be useful for immediately preserved specimens that are known to be pre-pupae, it cannot be safely applied to maggots that may have been feeding, and have been separated from food some time before preservation. Maximal potential for error in interpreting crop status would occur for starved “mid”-sized maggots because they are not fully grown, and may therefore paradoxically appear older than they are if mistaken for pre-pupae that have undergone post-feeding length reduction. This is complicated by the fact that degree of post-feeding shrinkage prior to pupariation is variable within species, making aging of pre-pupae inherently difficult [30]. For example, “mid”-sized *C. vicina* maggots were between approximately 13 and 15 mm by the end of the experiment, and were between approximately 5 and 6 days old depending on whether they were in the 24 h or 48 h treatment. However, 15 mm long *C. vicina* maximally shrunken pre-pupae take approximately 8 days to reach this stage if grown at 20 °C [28]. Also, the data of Anderson [31] reveal that the pre-pupal stage can be reached in roughly a minimum of 6 days, and end at roughly a maximum of 10 days at 20.6 °C. This gives potential for age overestimation of several days. Similarly, the data of Byrd and Butler [31] indicate that *Ch. rufifacies* grown at the slightly higher temperature of 21.1 °C would take roughly 120–160 h to reach the pre-pupal equivalent size of “mid”-sized larvae collected at the end of the experiment (10.7–14.5 mm), whereas the experimental maggots were only 120 h old at 20 °C prior to collection.

Predictions about the effect of evidential storage on “mid” and “large” larvae are further complicated by the findings of studies showing that the characteristics of the pupariation site material offered, or lack of a pupariation site, critically affects the length of the pre-pupal stage. This stage can be extended in situations where larvae of calliphorids or sarcophagids are exposed or waterlogged [30,32–34], which was true of the experimental setup here with larvae being kept well hydrated, and having only a piece of folded paper under which to hide.

It is counterintuitive that the 48 h fridge treatments for “mid” and “large” *Ch. rufifacies* maggots should have a slightly lower proportion of empty crops per treatment than 24 h refrigerated maggots. But these maggots came from different baseline stocks, which displayed some variation in their mean lengths (Table 3), and may therefore have emptied their crops at intrinsically different rates. The *C. vicina* stock sizes were more uniform in their growth, and logistics allowed use of common stocks for each size class because large numbers of individuals could be produced at one time. This is probably why the overall results for *C. vicina* generally follow clearer patterns. The measurements on each species were also carried out in different laboratories, limiting our ability to make definitive species comparisons. Nevertheless, it is still apparent that the crops of *Ch. rufifacies* are more likely to empty at room temperature, and also, when refrigerated, “small” maggots are more likely to empty their crops than “mid” and “large” maggots. Comparison with *C. vicina* reveals that refrigerated “large” maggots are much less likely to empty their crops than other treatments. The generally slower crop emptying seen in “large” refrigerated maggots of both species may therefore be indicative of different digestive rates between maggot age groups. This would make sense in the context of the rapid feeding and growth characteristic of maggots represented by the “small” and “mid” size classes of these two species [29,31,35], and the rapid gut transit times of 1–2 mm per minute that have been measured for actively feeding carrion fly maggots (species and age not given) [30].

The maintenance of developmental progression for “large” maggots of both species, despite evidential storage conditions, should come as no surprise. It is well understood that once third instar calliphorid larvae attain a sufficient size, they will leave their food source to pupariate [25,30], and it has also been shown for several calliphorid species that undersized maggots under strenuous food competition [36] or removed early from their food [37] still proceed to pupariation as long as they have reached a threshold size, which is as little as 45% of normal final weight for *Ch. rufifacies* [37]. This relates to the adequacy of fat reserves for making the transformation to adulthood, even though the resulting adult will also be undersized [30,37]. Ždárek [32] worked with underfed third instar *Sarcophaga bullata* Parker (Diptera: Sarcophagidae) larvae that had reached the pupariation threshold size. They had an extended pre-pupariation period in comparison to larvae fed *ad lib*. that were allowed to reach full size. The extension resulted because the underfed larvae did not lose their feeding instinct for between 30 and 40 h, which was interpreted as an adaptation to continue foraging for a time before giving up to pupariate. Similarly, “large” and perhaps even “mid”-sized *C. vicina* larvae are expected to have progressed to pupariation if they had been left for long enough. There were also very few deaths in the “mid”-sized *Ch. rufifacies* room temperature treatments, and a corresponding length decrease, indicating that they may also have been progressing towards pupariation. They were of adequate size to permit this according to Levot's results [37]. Indeed, many of the *Ch. rufifacies* larvae from the “large” 48 h room temperature treatment did pupariate during the experiment.

Widespread evidence of development, coupled with low mortality rates, was recorded for *C. vicina*, even despite refrigeration for half of the treatments, and lack of food for all of them. This shows that live evidential samples of all ages of *C. vicina* will tend to survive for at least 48 h, as long as they are kept well hydrated and aerated. The results also underline the cold adaptation ascribed to *C. vicina*, i.e. [21,35,38,]. The maggots used in this experiment were from a laboratory adapted population (acclimatized to 20 °C), but still endured sudden introduction into the refrigerator with low mortality and some continuation of their development. This shows that evidential *C. vicina* maggots collected even during warm times of the year are able to survive well under temporary refrigeration. It is also in keeping with the findings of others that un-acclimatized maggots of this species readily survive even longer periods of refrigeration at lower temperatures [20–22]. In fact, the larval stages of *C. vicina* are able to survive brief mean supercooling points of between −7 °C and −13 °C, despite being reared at 25 °C [38]. Additionally, Johl and Anderson [20] found no mortality at any growth stage resulting from chilling *C. vicina* reared at 24 °C for 24 h at 3 °C. Ames and Turner [23] confirmed that *C. vicina* larvae survive and continue to develop after chilling between 1 °C and 5 °C for 5 days when ordinarily reared at 20 °C (although mortality figures were not reported).

The same general hardiness under the conditions of this experiment was not seen for *Ch. rufifacies*, which almost all died in the “small” treatments and in the refrigerated “mid”-sized treatments. They also tended to lose length across treatments, and where mortality occurred, it is impossible to determine whether this shrinkage occurred pre- or postmortem. “Large”-sized larvae had the highest rates of survival; this may because they accumulated the largest metabolic energy reserves. This experiment also clearly showed that “small” *Ch. rufifacies* maggots will not survive long away from their food source, regardless of the temperature. Dehydration was not a factor, but possibly the younger larvae of this species are prone to starvation. High mortality under refrigeration is an expected finding because *Ch. rufifacies* is adapted to subtropical and tropical conditions [39], and has been suggested to need at least 15 °C ambient temperature in order to complete development [39,40].

Survival and growth in *Ch. rufifacies* did not appear to be improved by the high rates of cannibalism that especially occurred at room temperature, although other larvae could be hypothesized to form an important source of replacement food under starvation conditions. Far fewer larvae died overall in the “mid”- and “large”-sized room temperature treatments, so it is difficult to assess whether cannibalism provided an advantage to the survivors. It is also impossible to differentiate whether cannibalized larvae were consumed secondary to succumbing to natural causes or whether they were attacked and killed by conspecifics.

It is advised in the European Association for Forensic Entomology “best practice” guidelines that live samples be delivered to the entomologist within 24 h [5], and this is presumably because rapid delivery increases their chances of survival. The results of this study also support this recommendation because development in an unfed sample will be minimized if the entomologist has no choice but to use it for estimating the PMI_min_. Quick delivery will also help mitigate complications from changes in temperature experienced by specimens after their collection [27].

## Conclusion

Because development can potentially continue in live unfed maggot samples, it is not advisable to preserve them for the estimation of PMI_min_ if they have been stored for a period between their collection and receipt by the entomologist. There may be occasions where the entomologist has no choice but to use such live samples in estimating PMI_min_, but, if so, a caveat is needed in the forensic entomology report alerting the reader to the likelihood that some development has occurred. Storage of unfed maggot samples in this study led to length change (increase and decrease), crop emptying, moulting from the second to third instar and pupariation. However, because the pattern of degree and occurrence of these changes differed between species and size class, the result of storage is not entirely predictable.
